# Vision for Improving Pregnancy Health: Innovation and the Future of Pregnancy Research

**DOI:** 10.1007/s43032-022-00951-w

**Published:** 2022-05-09

**Authors:** James M. Roberts, Dominik Heider, Lina Bergman, Kent L. Thornburg

**Affiliations:** 1 Department of Obstetrics Gynecology and Reproductive Sciences, Magee-Womens Research Institute, Epidemiology and Clinical and Translational Research University of Pittsburgh, Pittsburgh, PA USA; 2 Faculty of Mathematics and Computer Science, University of Marburg, Marburg, Germany; 3 Department of Obstetrics and Gynecology, Institute of Clinical Sciences, Sahlgrenska Academy, University of Gothenburg, Gothenburg, Sweden; 4 Department of Obstetrics and Gynecology, Stellenbosch University, Cape Town, South Africa; 5 Department of Women’s and Children’s Health, Uppsala University, Uppsala, Sweden; 6 Departments of Medicine, Obstetrics and Gynecology and Biomedical Engineering, Bob and Charlee Moore Institute for Nutrition and Wellness, Knight Cardiovascular Institute, Oregon Health Sciences University, Portland, OR USA

**Keywords:** Preeclampsia, Innovation, Research, Training, Artificial intelligence

## Abstract

Understanding, predicting, and preventing pregnancy disorders have been a major research target. Nonetheless, the lack of progress is illustrated by research results related to preeclampsia and other hypertensive pregnancy disorders. These remain a major cause of maternal and infant mortality worldwide. There is a general consensus that the rate of progress toward understanding pregnancy disorders lags behind progress in other aspects of human health. In this presentation, we advance an explanation for this failure and suggest solutions. We propose that progress has been impeded by narrowly focused research training and limited imagination and innovation, resulting in the failure to think beyond conventional research approaches and analytical strategies. Investigations have been largely limited to hypothesis-generating approaches constrained by attempts to force poorly defined complex disorders into a single “unifying” hypothesis. Future progress could be accelerated by rethinking this approach. We advise taking advantage of innovative approaches that will generate new research strategies for investigating pregnancy abnormalities. Studies should begin before conception, assessing pregnancy longitudinally, before, during, and after pregnancy. Pregnancy disorders should be defined by pathophysiology rather than phenotype, and state of the art agnostic assessment of data should be adopted to generate new ideas. Taking advantage of new approaches mandates emphasizing innovation, inclusion of large datasets, and use of state of the art experimental and analytical techniques. A revolution in understanding pregnancy-associated disorders will depend on networks of scientists who are driven by an intense biological curiosity, a team spirit, and the tools to make new discoveries.

**Supplementary Information:**

The online version contains supplementary material available at 10.1007/s43032-022-00951-w.

## Introduction

### What Is Our Goal?

While death rates from heart disease in the USA have been decreasing across four decades, many diseases among women are on the rise. In the USA, maternal hypertensive disorders including gestational hypertension, preeclampsia, and eclampsia have nearly doubled in prevalence over the past three decades [1]. WHO data suggest that the incidence of preeclampsia may be up to seven times higher in low- and middle-income countries than in high-income countries [2]. These trends point to the sobering fact that medical science has not made substantial progress against this complex of pregnancy diseases despite valiant research efforts across the globe.

Given the upward trend in gestational disease rates, the medical community would benefit greatly by evaluating current obstetrical practices and then determining what progress they would like to see in terms of etiology, diagnosis, prognosis, management, and prevention of adverse pregnancy outcomes in the next quarter century.

Several important questions might focus our vision for understanding the pathophysiology of hypertensive diseases in pregnancy and the early diagnosis of these conditions as well as their treatment and prevention. Although we discuss hypertensive disease as an example, these considerations apply to all pregnancy research.

- What if we were able to predict vulnerability of hypertensive disorders of pregnancy before pregnancy?
- What if we could diagnose hypertensive disorders in the first weeks of pregnancy before manifestation of symptoms?
- What if we understood the pathophysiological processes that underlie preeclampsia in its various forms?
- What if we could recognize subsets of hypertensive disorders as unique pathophysiological variants of preeclampsia?
- What if we could recognize and categorize hypertensive disease subtypes and offer an evidence-based prognosis for the outcomes of the pregnancy and the long-term disease risks for mother and offspring?
- What if we could expand our therapeutic “toolbox” to safely manage compromised pregnancies that take dramatic turns mid-course?
- What if we knew enough about the risk factors for acquiring the disorders that we were able to offer strategies for prevention that were highly efficacious?

Once we, the scientific community who study preeclampsia and other hypertensive disorders, are able to envision the idea to which we aspire, we could develop strategies to pursue our utopian goal. The next step would be to think entirely out of the proverbial box toward innovations that could bring reality to future hopes. These innovations must necessarily link foundational knowledge to potential dramatic leaps that include all the resources that are available including biomedical engineering, nanotechnology, machine learning, artificial intelligence, all aspects of “omic” science, human psychology, epidemiology, mass education, and creative ways to secure a financial backbone for newly integrated science.

### What Is Slowing Progress? Is There a Solution?

What are the impediments to lightening-paced progress? Among medical scientists, there are two extreme camps that are harmful to the cause of scientific progress. On one hand, there are scientists who belittle the importance of scientific efforts unless it involves the use of new faddish technologies, many of which may be very promising. Scientists in this camp often downplay solid research that is making progress with well-established scientific techniques. On the other hand, there are scientists who are so tightly tied to the past that they do not offer hope for progress because of their unwillingness to recognize how modern technologies might benefit their own efforts to understand disease processes. Additionally, because the latter are often viewed as “fossils,” we have left behind their painstaking studies of pathophysiology that could bring new insight because their work does not seem to be on the cutting edge and does not get funded. Nevertheless, it is just as big a mistake to ignore exciting new fields that could make leaps forward in understanding disease processes.

We need to utilize the full power of current approaches, but it is mandatory to supplement them with innovative experimental and analytical strategies. Human physiology and pathophysiology are remarkably complex. The least complicated explanation for this complexity crudely states that it is the result of gene-environment interaction. However, it is not gene, but genes and not environment, but environments that must be considered. The modifications brought about by these interactions can be transient or permanent, stimulatory or inhibitory, or single or multiple. There are of course thousands of genes that affect the physiological environment and vice versa. In pregnancy, the interactions between genes and environments affect not one but two individuals. If we consider pregnancy disorders, the intricacies of these interactions are further magnified.

Over the past century, these complexities were recognized but to a large extent deciphering them was beyond conventional research strategies. Because of this, biological research adopted the Occam’s razor approach, looking for the least complex explanation for biological questions. Furthermore, hypothesis-driven research was mandated, and investigators were encouraged to study in great depth a narrow slice of pathophysiology with the belief that somehow, some way, some day we would put the slices together.

With the advent of modern computing and bioinformatics, the possibility of an agnostic approach to understanding the big picture became possible. With the power of current computational algorithms, it is possible to identify patterns, clusters, and interactions without preconceived hypotheses. This power is manifested in the analytical strategies of machine learning and artificial intelligence. These approaches use algorithms that allow data analyses of analytes formerly considered not particularly revealing to now provide valuable insights. In addition, they reveal relationships that have not been imagined by hypothesis-driven studies.

Currently, the major impediment to exploiting the use of these new strategies is that they are not well understood and thus are not being used. Part of this is because investigators do not use what they cannot understand. In this presentation, we remedy this to some extent by considering the strengths and limitations of artificial intelligence. The other challenge to the use of these approaches is the requirement for large amounts of data including excellent clinical data. When these limitations have been overcome, we will finally begin to unravel the complexity of human disease including the most complex of all, pregnancy disorders.

In this presentation, we consider innovative experimental strategies to complement conventional approaches and current analytical strategies that can provide new insight into both old and new approaches by agnostic approaches to data analyses. We also describe the challenges provided by such analyses and how these might be addressed. 

## Innovative Approaches to the Study of Adverse Pregnancy Outcomes 

### The Standard Approach to Pregnancy Research

The standard approach to pregnancy research is typically intervention or observation at one or a few times in pregnancy, and registration of outcomes after delivery and in some cases months or years postpartum. Traditional research is hypothesis driven with a deliberately chosen narrow focus. The advantage of this approach is that it is well-established and connected to possible windows of intervention and follow-up. It is also tailored to fit with current pregnancy databases and registries in which information about the pregnancy is typically collected in the first or second trimester at the woman’s first visit to the antenatal clinic. Outcomes are typically collected after birth from medical charts and in national and quality registers. “Focus” is celebrated with the concept that eventually the “small slices” of pregnancy physiology and pathophysiology will be pieced together to a meaningful whole.

The disadvantages of this standard approach are that the data used for analysis are usually limited to what is registered in a trial or at pre-defined time points that are often few and far apart. They sample little of the dynamically changing events of pregnancy. Data are usually collected when women present for care, rarely earlier than 6 weeks of gestation (menstrual age). Thus, we know little about the early adaptations of pregnancy. It is also increasingly appreciated that events prior to pregnancy (e.g., decidualization and receptivity of endometrium [3]) may strikingly modify later pregnancy events. There is currently little evidence that identifies these relationships. Longitudinal research is rare and collection at more than a few time points unusual. The hypothesis-driven research also limits the ability to discover new associations. This standard approach to research has been successful to advance understanding. However, there are tremendous gaps in our knowledge that will not be filled until we begin to radically modify conventional strategies with alternative innovative approaches.

### Alternative Approaches to Pregnancy Research

#### Longitudinal Approach

Since pregnancy is a time of rapid physiological changes, it is likely that changes in measurements over time would be more predictive of disease and better define pathophysiology than assessment at one time point only. This has been challenging to achieve with the usual design of current research studies. Acquiring measurements at several time points requires that the pregnant woman is contacted or scheduled for follow-up several times during pregnancy. This is time consuming and expensive with the addition of resulting loss to follow-up. Traditionally, research studies that have been designed to require as few contact points as possible have been more successful due to the above issues. However, with rapid advancements in technology, we have the opportunity to follow research participants more closely and at multiple time points with much less impact on subjects.

#### Wearables for Continuous Longitudinal Data Analysis

The use of “wearables,” devices which can continuously and noninvasively monitor physiological processes or behavior, has the capacity to make longitudinal data analysis possible. One example of this is the use of portable pollution monitors. The role of environmental pollutants to adversely affect normal pregnancy is well established. The approach to acquiring information to support this conclusion has characteristically involved the use of zip codes to establish residency location combined with measurements from neighboring environmental pollution stations [4]. The pollution measured by these stations is combined with the geographical relationship of the residence to the pollution station from which environmental contamination measurements are calculated. These measurements assume the woman is at her home constantly through her entire pregnancy which is, of course, far from reality. In recent studies, a portable monitoring device has been used to determine exposure to pollutants over prolonged periods [5]. With this information, a much more accurate determination of environmental exposures is possible. Similar wearable devices for measuring heart rate, blood pressure, activity, etc. are available or in development, allowing the longitudinal and prolonged assessment of physiological processes. Some of these are quite innovative. The use of transdermal optical imaging [6] with a standard smartphone camera to measure facial skin blood flow uses artificial intelligence analysis to convert these data for blood pressure [7], heart rate, and heart rate variability analyses. By combining these measurements, the device can also assess stress levels [8]. This technology has also been used to measure glycosylated hemoglobin including HbA1c [9], and there are plans to design an app with the capability of measuring blood lipids [6].

Even with the present technology, the GPS that is embedded in virtually all smart phones or other mobile devices enables researchers to evaluate distances covered daily and the location of potential exposures [10, 11]. In addition, by increasing the use of smartphones, research participants can easily be followed through applications customized to a study in which participants can enter data weekly daily or even several times daily data. Examples of this are assessment of dietary habits, exercise, current symptoms, and other lifestyle factors such as smoking or alcohol use [12, 13]. Current technology can collect and interpret longitudinal data obtained from pregnant women using smart phone technology. Such data could be important for quantifying and understanding changes of exposures during the course of pregnancy.

#### Assessment Before and in Very Early Pregnancy

There are several well-established pathologies and metabolic conditions that increase the risk of preeclampsia [14]. These could provide hints about the pathophysiology of preeclampsia and other pregnancy disorders. Most current thinking associates these pre-pregnancy factors with later pregnancy events suggesting they “sensitize” the woman to the pathophysiology of preeclampsia. However, it is becoming evident that pre-pregnancy changes such as the formation of receptive decidua [15] and a functioning corpus luteum [3] and very early pregnancy changes [16] are extremely important for normal pregnancy. This makes it likely that some degree of “sensitization” occurs before or in very early pregnancy. Unfortunately, pre-pregnancy and very early pregnancy data relevant to adverse outcomes are extremely scarce in current research. In addition, not all women wish to become pregnant, and limiting pre-pregnancy studies to only women planning pregnancies results in findings that may not be generalizable. In theory, electronic medical records (EMR) could allow assessment before pregnancy in many women, though limited to women seeking health care. In addition, it also provides substantial challenges because of the wide varieties of EMR and the fact that in most settings the instruments are better designed for billing needs rather than clinical or research uses [17]. The wide availability of smartphones, the emerging availability of non-invasive physiological assessment, and the simplicity of intermittent reporting provide potential solutions to this problem.

The power of smart phones to not only monitor but to modify behavior is indicated by studies with an app developed to modify at-risk behaviors before and during pregnancies [13]. In a study of women presenting for IVF, subjects received an app to modify behavior (Smart Pregnancy) accompanied by on-line coaching before and during pregnancy. In this highly motivated group, compliance was 75% and the intervention was successful to improve dietary intake and reduce risky behaviors (smoking and alcohol intake). The success with this approach and especially with compliance provides an encouraging indication that women can be identified and recruited before pregnancy, which in addition to providing help for the woman for her own use, can also provide valuable information for the research team.

#### Life-Course Studies

It is increasingly evident that events encountered during pregnancy are determinants of later life health [18]. Women with pregnancy disorders including preeclampsia [19, 20], delivering preterm [21], or women with pregnancies complicated by fetal growth restriction [22] are associated with increased risk of cardiovascular disease later in life. Even having several normal pregnancies is suggested to increase risk of cardiovascular disease (CVD) [23]. Conversely, at least with a single normal pregnancy, there is a reduction in later life CVD [24] and an improvement in cardiovascular function for at least 1 year [25].

Most studies of how pregnancy affects health later life health are directed at pregnancy abnormalities. It is likely with this approach that medical scientists are missing an opportunity to identify other important relationships. It is quite possible that failed pregnancy adaptations that do not cross a threshold to result in adverse pregnancy outcomes are also associated with increased or reduced later life disease.

It is vitally important that pregnancy and its outcomes should be considered as an integral component of the life course. With adequate data, it would be possible to determine whether CVD in later life provides insight into the pathophysiological underpinnings of preeclampsia. Could the later life development of CVD in a woman who once had preeclampsia identify a different underlying pathophysiological pathway than would be present in a woman with preeclampsia who did not manifest CVD in later life [26]?

Testing these associations demands better life course data than are currently available. Registry-based data hold important information about exposures and outcomes over a long period of time, sometimes over the span of a lifetime. As an example, national registers of pregnancy and childbirth in Scandinavian countries date back to the 1960s or 1970s, meaning that pregnancy exposures can be followed and investigated for different outcomes 50 years after childbirth [27]. These epidemiological studies originating from national registers are confined to a few countries. However, even the availability and use of such registries leaves large gaps in our knowledge. At best, a study shortly after pregnancy is linked with outcomes decades later with little or no information in the intervening years. It is encouraging that studies are now in progress of women entered into cardiovascular assessment prior to reproduction who are now being followed for the relationship of CVD to pregnancy outcome [28]. However, these studies were not designed for this purpose and pregnancy information is limited. Another step forward is the follow-up of women with pregnancy outcome data and biological samples obtained during pregnancy [26]. This is a valuable resource but again, samples and data were repurposed for CVD follow-up with modest CVD data obtained during and none before pregnancy.

Ideally, what is needed is the assembly of a diverse pre-pregnancy cohort to be followed from before pregnancy until death. Although such a study would be expensive and difficult, the information gathered on pregnancy and life course would be invaluable.

#### Discovery-Based Research

As mentioned above, hypothesis-based research has been considered the most appropriate strategy to identify pathophysiological pathways and associated biomarkers. The availability of large datasets and powerful bioinformatic techniques (see below) now allow agnostic, hypothesis-generating approaches. This alternative approach to the study of diseases identifies clusters of analytes (proteins, metabolites expressed RNA, etc.) and with systems biology seeks the identity of relevant pathophysiological pathways [29]. This approach could reduce heterogeneity in pregnancy-induced disorders such as preeclampsia and preterm birth, identifying subtypes of the disease and improving detection rates of diagnostic and prognostic tests and directing appropriate therapy [30]. An example of this was provided by McElrath and his research group who utilized proteomics and genomics from samples early in pregnancy that could phenotype later preeclampsia by subtype (severe/non-severe, biochemical abnormalities, and gestational week at delivery) [31]. The power of this approach is described below.

#### Resilience

The classical approach in research has been to detect disease, focusing on the women diagnosed with various complications and to compare them with healthy controls. An emerging new approach is to investigate resilience, or why some women do not become ill even though carrying several risk factors. By identifying protective factors such as protein expression or protective life-style factors such as diet, researchers can identify new targets for intervention, particularly in high-risk women [32, 33].

An example of resilience would be a woman with several strong risk factors for preeclampsia (such as twin pregnancy, systemic lupus erythematosus, or previous early onset preeclampsia) that goes through pregnancy without evidence of the disease. What are the underlying factors that define her phenotype? Which factors are protective against preeclampsia? Such information can provide extremely useful for future preventative targets in pregnancy.

#### Pregnancy Abnormalities Defined by Pathophysiology

**Fig. 1:**
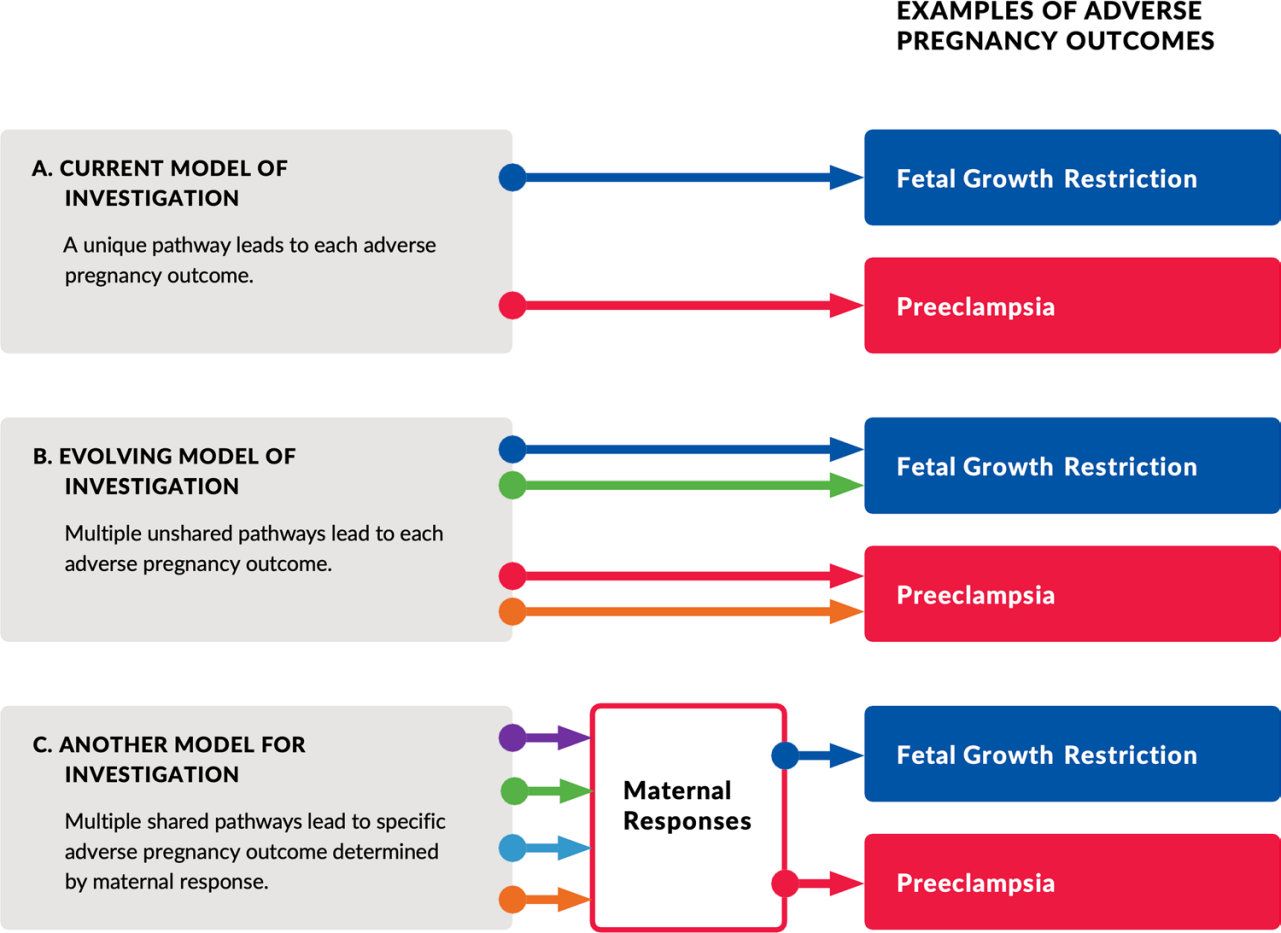
Hypothetical pathophysiological pathways to adverse pregnancy outcomes: Panel A indicates the conventional hypothesis that adverse pregnancy outcomes (here represented by preeclampsia and fetal growth restriction) each has a distinct, single pathway. Panel B presents an evolving hypothesis that there are several pathways to adverse outcomes with different pathways to each outcome. In panel C, an alternate hypothesis suggests there are multiple pathways to adverse outcomes, and these are shared by all outcomes. The particular adverse outcome is determined by the maternal response to the insult (e.g., the same insult in different women can lead to preeclampsia or fetal growth restriction). For simplicity, pathways are shown as independent but it is likely there are complex interactions between pathways

Pregnancy-related disorders are commonly diagnosed by criteria based upon phenotype (e.g., preterm labor, fetal growth restriction, preeclampsia). It is interesting that these diverse phenotypes seem to share common pathophysiological features including placental pathological [34], metabolic [35, 36], and inflammatory [35, 36] features. They also have common epidemiological risk factors and a similar relationship to later life CVD [22]. This raises the possibility that these different phenotypes might share subtypes with similar pathophysiological pathways to disease but different maternal response (Fig. 1). For these disorders, it is likely that there are several and similar pathophysiological pathways to these endpoints. This is especially evident with preeclampsia which can present with radically different clinical, laboratory, and pathological findings, and relationship to later life disease [37]. Although not as well appreciated, a similar diversity is equally likely with preterm birth and fetal growth restriction. With current discovery science, it should be possible to divide these diagnoses into subgroups based upon underlying pathophysiology. For example, preeclampsia is posited to be the result of a poor placentation with resulting syncytiotrophoblast stress [38]. There are, however, likely several pathways to abnormal placentation (immunological, toxins, etc.) [30]. Alternatively, preeclampsia could occur with normal placentation where the woman from prior to conception demonstrates an injured endothelium secondary to diseases such as obesity, inflammatory disease, or hypertension [39]. Furthermore, syncytiotrophoblast stress can be the result of phenomena other than abnormal placentation (e.g., infection, placental senescence) [38]. These alternative pathophysiological pathways need different treatment and are associated with different biomarkers. Research utilizing current discovery strategies directed to pathophysiological pathways to preeclampsia and other pregnancy-related disease could potentially achieve directed detection and treatment.

#### Designing Research Studies to Meet the Needs of Low Resource Settings 

There is an enormous imbalance in allocation of research funds and research activity in obstetric research between low-middle-income countries and high-income countries. The vast majority of resources are spent in high-income countries whereas the need for discovery and implementation is much larger in low-middle-income countries. Often, proposals that require new technology and resources exclude innovative research in low-middle-income countries. We argue for the opposite — new technology can facilitate research and health care improvement in low-middle-income countries, to a higher degree than for high-income countries. An example of using advanced technology in pregnancy research is the use of artificial intelligence to interpret a digital image of a placenta [40]. By obtaining a picture of the placenta, in a standardized manner, the interpretation using AI techniques can then correlate the picture with [15] diagnoses such as abruptio, chorioamnionitis, and marginal insertion of the umbilical cord and perhaps uncover other morphological association with pregnancy outcomes. AI techniques have the possibility to be translated also into other areas such as ultrasound images or other clinical signs and could standardize and improve care for pregnant women worldwide.

## Innovative Analytical Strategies

### Introduction

The field of artificial intelligence (AI) combines computer-based algorithms and robust datasets to solve problems not otherwise solvable. A number of sub-fields of AI in biomedicine have emerged and include interrelated unsupervised machine learning for clustering of data, e.g., subtyping, and supervised learning, e.g., deep learning and neural networks, for diagnostics and prognostics. These sub-disciplines are based upon AI algorithms which are able to solve complex problems, discover previously unknown relationships or groupings, and make predictions or classifications derived from input data. The language of AI is unfamiliar to most reproductive scientists and clinicians, but the terminology becomes clear as the concepts are examined.

Table 1 contains the definitions of the most commonly used terms.

**Table 1:** Definitions of the most commonly used terms

Term	Explanation
Artificial intelligence (AI)	AI refers to modern technologies able to learn patterns or functions from data that can be used to predict new, unseen data, e.g., for diagnostics
Machine learning (ML)	ML is used synonymously with AI; however, historically AI is broader than ML
Clustering	Type of unsupervised learning where data is grouped together based on a mathematical similarity
Deep learning (DL)	DL refers to deep neural networks, i.e., a special type of ML architecture that is based on many neurons arranged in layers. There are many types of DL architectures; however, feed-forward neural networks are the most common
Euclidian distance	Distance metric
*k*-means	Clustering algorithm
*k*-medoids	Clustering algorithm
Linear regression	Simple statistical method for modeling the relationship between metric variables
Linear problem	Classification or regression problem that can be solved by a linear model, e.g., a hyperplane
Non-linear problem	Classification or regression problem where the data is non-linearly distributed, e.g., polynomially
Logistic regression (LR)	Simple statistical model for binary classification problems and statistical inference
Neural network (NN)	NN are ML models that are based on networks (or graphs) of neurons able to process information and to find an abstract representation of the data
Neuron	Simple ML model (also called perceptron) that is able to solve linear problems. Basic node in an NN
Feed-forward neural network	Special type of NN where information is only transferred from one layer to the next without any loops
Supervised learning	ML models are trained on data that has different classes, e.g., case and control
Unsupervised learning	ML models are trained solely on the data without any label information. The data is clustered into groups

**Fig. 2:**
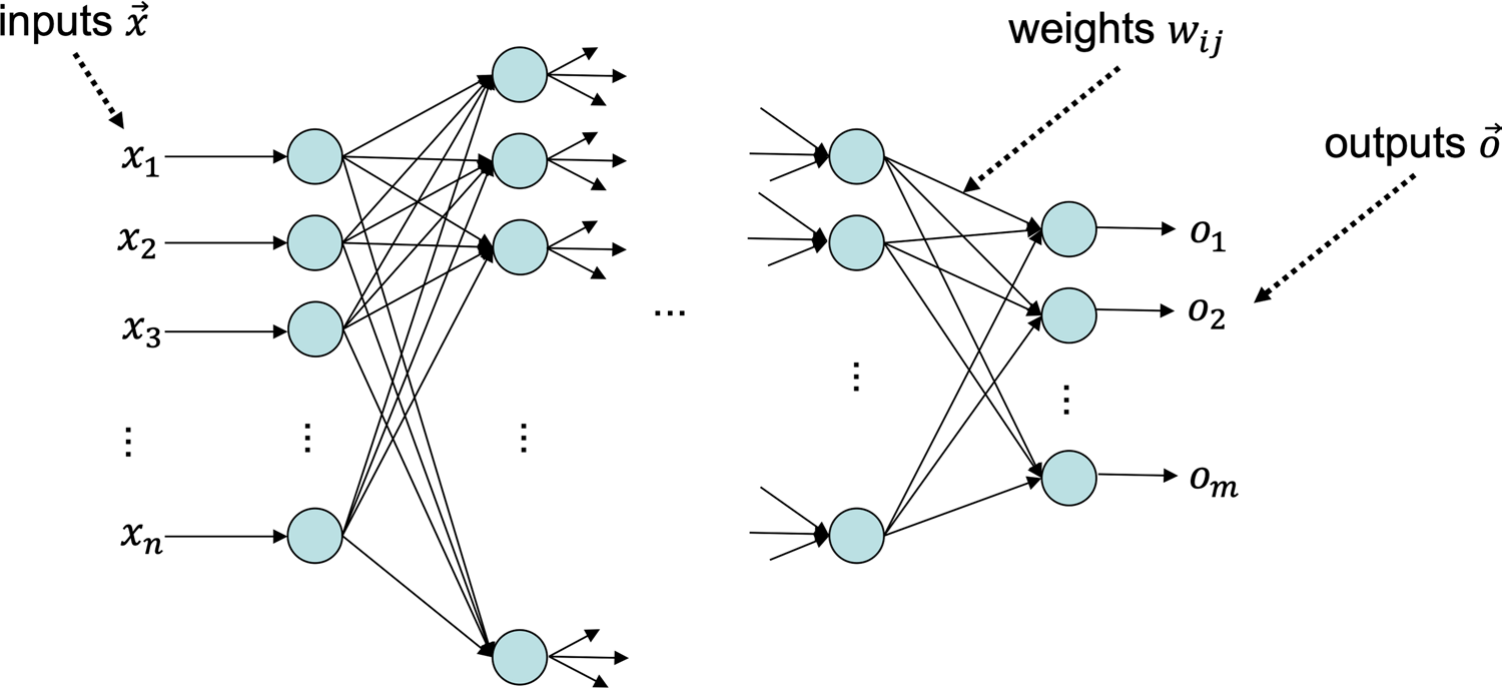
Schematic illustration of a feed-forward neural network: Neurons are shown in light blue. The input layer is shown on the left, the output layer on the right. In between, there can be several hidden layers with an arbitrary number of neurons

Deep learning is a sub-field of machine learning which features “neural networks.” The idea of a neural network is not necessarily related to neurons in the brain but to the idea of interacting nodes that operate in layers. The depth of deep learning relates to the number of layers in a neural network. See Fig. 2.

Machine learning and artificial intelligence are being used in many areas in medicine and have been recently reviewed; examples include oncology [41], pathology [42–44], diabetes [45, 46], human genetics [47], and infectious diseases [46, 48, 49], as part of a growing trend toward personalized/precision medicine. However, these approaches are currently underrepresented in pregnancy research. The reasons are manifold but are mainly because standard approaches, such as biostatistics, are often included in educational programs for trainees, while machine learning is not. However, machine learning for clinical diagnostics can offer many advantages compared to standard statistical approaches. For instance, while statistical approaches such as linear or logistic regression are widely used, these approaches are only able to capture linear associations between the variables and observations. Modern approaches from the field of artificial intelligence, such as deep learning, can also model non-linear relationships that are present in real-world problems. Moreover, in contrast to classical statistical approaches, modern machine learning approaches are based on optimization techniques, thus not guaranteeing an optimal solution. Although this sounds like a disadvantage of machine learning algorithms, it is actually an advantage as these techniques can handle large amounts of data combined with non-linear models resulting in valuable but unanticipated results. However, this does not come without a cost. Many machine learning approaches, such as deep learning, are considered as “black boxes,” and understanding the causal relationships for an outcome is often hidden. This fact makes AI approaches uncomfortable for clinical investigators who are not trained in its use, and the outcome may be difficult to interpret. While technical limitations, such as computing power, were once seen as a huge disadvantage of machine learning techniques such as deep learning, the availability of computing resources and centers as well as graphics processing units (GPUs) has greatly reduced this disadvantage. However, using AI for medical purposes poses additional problems. For instance, regulatory requirements for the use of AI software as a medical device are not yet fully clarified.

### Demystifying AI

Machine learning models can be roughly separated into three categories, namely supervised, unsupervised, and reinforcement learning. While supervised and unsupervised learning are typically used in medical settings, reinforcement learning is rather a niche and not widely applied. Thus, we will concentrate upon supervised and unsupervised learning.

In unsupervised learning, the goal is to identify subgroups in a dataset, in which each sample (e.g., patient cases) shares some similarity with the other samples in the subgroup and differs from the other subgroups. This task can be solved by using clustering algorithms, which can be separated into partitional and hierarchical clustering. The idea of hierarchical clustering is that we try to find clusters in a top-down or bottom-up approach. In the top-down approach, the so-called divisive hierarchical clustering, all samples are put into one cluster which is then split into smaller clusters in an iterative manner. In contrast, the bottom-up approach, the so-called agglomerative clustering, starts with *n* clusters for *n* samples (i.e., one sample per cluster) and tries to merge smaller clusters to bigger ones. Partitional clustering, on the other hand, splits the data into a predefined number of clusters by using a specific algorithm. The most prominent algorithms for partitional clustering are *k*-means and *k*-medoids (Coombes et al., 2021). In the *k*-means algorithm, *k* cluster centers are randomly initialized (i.e., random coordinates) and all samples in the dataset are assigned to one cluster center, namely to the one that is the closest, using a distance function, e.g., the Euclidian distance. After these assignments, the cluster centers are updated. The new cluster center is the arithmetic mean of the samples within the cluster. This procedure is repeated in an iterative manner until all samples are stably assigned to their clusters. Because *k*-means is very unstable with respect to its initial cluster center position, as these initial coordinates are random, the *k*-medoids algorithm uses existing, random samples as centers instead. Unfortunately, there is no clustering technique that is superior on all datasets; thus, it is important to compare the performance of the different algorithms for the specific dataset at hand. Examples for the use in clinical research include, for instance, the identification of disease subgroups, e.g., preeclampsia subtypes, or patient stratification [31].

In supervised learning, the goal is to find a function *f* that maps the independent variables in a dataset onto a class label, the dependent variable. The most common case is that the class label is a dichotomous variable and can be either positive (e.g., case) or negative (e.g., control). Typically, positive and negative are represented as 1 and 0. Depending on the algorithm at hand, finding the function *f* can be seen as an optimization problem. There are many algorithms available for this task, e.g., from the field of statistics, such as logistic regression, toward more modern machine learning approaches, e.g., support-vector machines, random forests [50], or deep learning. As an example, let us consider a neural network (NN). An NN is basically a graph of nodes (here called neurons) and edges (i.e., connections between these neurons), which is set up in layers (see Fig. 2). Please note that, for simplicity, we consider a classical feed-forward network in this example; however, other types of topologies are also possible and used in practice. In the first layer, the inputs are collected and forwarded into the network. These inputs could be, e.g., clinical parameters, such as blood parameters, demographics, e.g., age and gender, or also imaging or omics data. Depending on what data are used, in particular for omics and imaging data, the data need to be preprocessed before use.

The processing of clinical data for machine learning is typically separated into different steps, namely (i) data cleaning (e.g., outlier detection, missing values), (ii) training of a neural network, and (iii) validation of the model. The training of a neural network is typically done by an algorithm called Backpropagation, which is based on a gradient descent approach. For detailed information, see Supplement.

As clinical datasets are typically rather small, preprocessing not only includes missing data imputation, i.e., estimating or predicting the missing values, but also reduces the parameters to those that are essential for the classification problem at hand. This is not only important to overcome the so-called small-*n*-large-*p* problem, but also to reduce costs in clinical practice. The small-*n*-large-*p* problems refer to the difficulty that for modeling problems with a small number of samples *n* (here patients and controls) and parameters *p* (here, e.g., blood parameters), machine learning models tend to overfit the data, i.e., they perform well on the training data, but poorly on the validation data. Feature selection methods (also called biomarker discovery) aim at reducing *p* by selecting those parameters that are relevant (from a computational point of view) for the prediction problem at hand. There are many different examples of feature selection approaches used in the literature; however, it has been shown that combining these methods into ensembles gives more reliable selections [51].

Furthermore, clinical decision-support systems need to be probabilistically interpretable, typically addressed by calibration methods [52], and need to be balanced by data augmentation techniques [53], and uncertainty [54] in the classification process should also be considered to improve acceptance by the medical community and patients.

### Machine Learning for the Study of Preeclampsia

Once again, taking preeclampsia as an example of an adverse pregnancy outcome, the use of machine learning has just begun to be used. The results, nonetheless, are very exciting. In a study of the relationship of diet to preeclampsia and other adverse pregnancy outcomes, machine learning was compared to traditional analyses to test the relationship between diet and adverse outcomes. In the traditional analysis, there was a weak relationship between nutritional findings and preeclampsia but to no other adverse pregnancy outcomes. However, a machine learning strategy which permitted the evaluation of nutritional synergies demonstrated relationships between nutritional factors and not only preeclampsia but also fetal growth restriction, preterm birth, and gestational diabetes [55]. Similar improvements have also been demonstrated for preeclampsia prediction using routinely collected clinical data [56] which has been remarkably effective in this and other studies [57, 58]. These early studies demonstrate the power of these new analytical strategies.

### Limitations of AI and Potential Solutions

There are, however, limitations for the use of AI in women’s health, and in medicine in general. First, as addressed previously, AI and machine learning are currently not part of the curricula in most medical departments. Thus, acceptance by medical experts is currently limited. This is particularly true for models such as deep learning, as they are seen as black boxes, and interpretation of the predictions, in particular why and how a decision has been made, is partly unsolved. Moreover, data sharing is still an issue with respect to data protection regulations, which presently limit the use of machine learning in clinical practice. Moreover, regulatory aspects of AI in medicine are currently not well defined, which is, however, already on the agenda of regulatory agencies such as the Food and Drug Administration (FDA) or the European Medicines Agency (EMA). While some of these limitations need to be addressed by changes in the curricula and political and regulatory frameworks, others can be solved solely with technical approaches.

AI offers an enormous potential for improving and revolutionizing many areas in medicine, e.g., by providing personal diagnostics or therapy recommendations. Modern approaches of AI, e.g., federated machine learning or transfer learning, which can also handle small data sizes thus allowing collaborative, multi-center studies beyond national borders.

AI approaches for medical diagnostics must fulfill data privacy and data protection regulations and data sharing, in particular between different countries which can be difficult. Several approaches have been proposed to satisfy many aspects of these regulations. In particular, federated learning has been identified as a promising candidate. Federated learning does not need to share data between different parties (Yang et al., 2019). Instead of sharing data between different parties, machine learning models are trained at each partner site based on local data. These models are then combined, thereby improving overall predictive performance while the data itself remains private. The most common approach for federated learning is horizontal learning where different models are trained on different local datasets and subsequently aggregated. In contrast to horizontal federated learning, vertical federated learning combines models with different features but identical samples.

Taken together, AI has a huge potential for data analytics and ultimately novel, personal diagnostics in pregnancy research. To improve acceptance by physicians, machine learning should be part of teaching curricula to enable a transition to a new era of digital medicine.

### The Challenge of Harmonized Data

Machine learning benefits from large amounts of well collected data. Characteristically, large data for pregnancy studies come from administrative data (e.g., hospital admissions and discharges and birth certificates). These data are limited by inaccuracy and deficits in the data collected. As discussed, EMR have the potential to provide detailed research information but are not standardized and in most cases were not designed for research usage. There is a rich resource of detailed pregnancy data present in data collected for research studies. Currently, with some exceptions, most of these data are not available for data sharing. Part of this may be due to the reluctance of investigators and their institutions to share data [59]. However, the major impediments to sharing are non-standardized terminology, dissimilarity in data fields and outcomes acquired, and incompatibility of databases that are rarely designed for sharing. Several funding agencies are encouraging sharing of data, getting around the first impediment. There are also beginning efforts to standardize data fields [60] and outcomes [61, 62] collected. All of these improve the data to be shared but there is little being done to harmonize the data collected. The idea of collecting data in a manner that facilitates sharing has not been widely accepted. It seems that carefully collected data are thus being wasted in many studies. Efforts to provide standardized databases have been met with some resistance by investigators reluctant to modify their personal data collection tools [63]. It would seem a reasonable solution would come from funding agencies, who similar to invoking open access to publication as a requirement for funding might use a similar strategy to mandate the use of harmonized databases.

## Summary and Recommendations

In the opening paragraph, we offered bulleted hypotheticals as goals for improving prevention, diagnosis, and treatment of women during pregnancy. Making progress toward those goals requires a new way of thinking about the complexity of disease through cutting-edge innovation based on carefully acquired data from hosts of women. The primary barrier to innovation in health care for women is the glacial progress in our understanding of adverse pregnancy outcomes. The good news is that there are exciting new tools available to overcome this inertia. The question remains: What can be done to make this happen?

Progress in understanding hypertensive disorders of pregnancy and other detrimental conditions demands a change in mind set to widen our thinking. We must avoid trying to force adverse outcomes into explanation by a single hypothesis. These conditions are largely syndromes defined by common phenotype. As we increase our understanding of the physiology of pregnancy and the pathophysiology of these disorders, their inherent complexity demands that we begin considering that different pathological pathways can lead to a common phenotype. We should also consider that the different phenotype defining a specific adverse outcome may share a common pathophysiology interacting with a different maternal response to the insult (Fig. 1). We should celebrate rather than ignore outliers. It is also clear that the use of machine learning tools requires large amounts of carefully collected data that can only be acquired by data sharing. Similarly, the complexity of diseases of pregnancy demands intellectual collaboration by individuals with diverse expertise. As part of widening the thinking of investigators, we must begin to appreciate and reward innovation. With limited available funding, it is the tendency of investigators, funding agencies, and reviewers to avoid risk taking and fund what has been shown to be successful in the past. Somehow, even though funding is limited, we must determine a way to reward well-conceived risk in research, which is, of course, a key component of innovation. Without the opportunity for clinicians and scientists to become educated regarding the utility of AI in many areas of women’s health, these new fields will not be embraced by these professionals. Thus, it will be important to develop curricula for trainees to become familiar with the techniques and to incorporate experts inside professional circles.

### Recommendations

1. Training
  - Innovation, which is actually teachable [64], must become a component of all training programs.
  - We must emphasize that even the most exciting hypothesis will not completely explain adverse pregnancy outcomes. Thus, data must be analyzed and presented in a manner which shows variability rather than obscuring it. We should teach trainees to celebrate rather than ignore outliers to encourage investigating new pathways.
  - We should bring “fresh eyes” to our studies by encouraging interactions with investigators from other disciplines, Thus, success demands collaboration with other investigators in different disciplines *which should be a focus of training*. Included in this should be attempts to begin to unravel the jargon which characterizes all disciplines.
  - It should be compulsory for trainees to attend presentations beyond their own field of expertise.
  - There should be training to understand the principles of the latest analytical approaches to allow “fearless” use of these strategies.
2. Innovative research
  - Innovative research should be given more than lip service as a component of fundable research.
  - Innovation should be considered highly important for academic promotion.
3. Interdisciplinary research
  - This is required to maximize progress in understanding complex diseases. It should include a broad array of disciplines: artificial intelligence, big data, informatics, new statistics, engineering, big databases, data-sharing, etc.
4. We should begin to extend “pregnancy” research to include pre-pregnancy, very early pregnancy, and post-pregnancy (short and long term) events using innovative approaches.
5. We should ensure that we use up-to-date approaches for data analysis guided by analytical experts.
6. Successful data sharing demands the use of harmonized databases. This should be considered as a requirement by funding agencies. It is clear we now have analytical tools to better understand disorders as complex as adverse pregnancy outcomes. Our goal now is maximizing our ability to exploit these. This demands innovation, interdisciplinary research, and data sharing. These strategies must be part of our research armamentarium and that of future investigators if we are to accelerate our understanding of pregnancy and associated adverse outcomes.

## Supplementary Information

Below is the link to the electronic supplementary material.

Supplementary file1 (DOCX 16 KB)

## Data Availability

Not necessary (review).

## References

[1] CDC. Data on selected pregnancy complications in the United States. 2019; https://www.cdc.gov/reproductivehealth/maternalinfanthealth/pregnancy-complications-data.htm#hyper.

[2] Wagnew M, Dessalegn M, Worku A, Nyagero J (2016). Trends of preeclampsia/eclampsia and maternal and neonatal outcomes among women delivering in Addis Ababa selected government hospitals, Ethiopia: a retrospective cross-sectional study. Pan Afr Med J.

[3] Conrad KP (2020). Evidence for corpus luteal and endometrial origins of adverse pregnancy outcomes in women conceiving with or without assisted reproduction. Obstet Gynecol Clin North Am.

[4] Lee PC, Roberts JM, Catov JM, Talbott EO, Ritz B (2013). First trimester exposure to ambient air pollution, pregnancy complications and adverse birth outcomes in Allegheny County. PA Matern Child Health J.

[5] Bui AAT, Hosseini A, Rocchio R (2020). Biomedical REAl-Time Health Evaluation (BREATHE): toward an mHealth informatics platform. JAMIA Open.

[6] Barszczyk A, Zhou W, Lee K. AIM and transdermal optical imaging. In: Lidströmer N, Ashrafian H, eds. Artificial intelligence in medicine. Cham: Springer. 2021.

[7] Luo H, Yang D, Barszczyk A (2019). Smartphone-based blood pressure measurement using transdermal optical imaging technology. Circulation Cardiovascular imaging.

[8] Wei J, Luo H, Wu SJ, Zheng PP, Fu G, Lee K (2018). Transdermal optical imaging reveal basal stress via heart rate variability analysis: a novel methodology comparable to electrocardiography. Front Psychol.

[9] Salih H, Wu SJ, Kabakov E, Lee K, Zhou W (2021). Smartphone-based identification of critical levels of glycated hemoglobin A1c using transdermal optical imaging. UTSC Journal of Natural Science.

[10] Shelton J, Casey S, Puhl N, Buckingham J, Yacyshyn E (2021). Electronic patient-reported outcome measures using mobile health technology in rheumatology: a scoping review. PLoS One.

[11] Michard F (2021). Toward smart monitoring with phones, watches, and wearable sensors. Anesthesiol Clin.

[12] van Dijk MR, Koster MPH, Oostingh EC, Willemsen SP, Steegers EAP, Steegers-Theunissen RPM (2020). A mobile app lifestyle intervention to improve healthy nutrition in women before and during early pregnancy: single-center randomized controlled trial. Journal of Medical Internet Research.

[13] Oostingh EC, Koster MPH, van Dijk MR (2020). First effective mHealth nutrition and lifestyle coaching program for subfertile couples undergoing in vitro fertilization treatment: a single-blinded multicenter randomized controlled trial. Fertil Steril.

[14] Burton GJ, Redman CW, Roberts JM, Moffett A (2019). Pre-eclampsia: pathophysiology and clinical implications. BMJ.

[15] Conrad KP, Rabaglino MB, Post Uiterweer ED (2017). Emerging role for dysregulated decidualization in the genesis of preeclampsia. Placenta.

[16] Huppertz B (2008). Placental origins of preeclampsia: challenging the current hypothesis. Hypertension.

[17] Dean BB, Lam J, Natoli JL, Butler Q, Aguilar D, Nordyke RJ (2009). Review: use of electronic medical records for health outcomes research: a literature review. Med Care Res Rev.

[18] Roberts JM, Hubel CA (2010). Pregnancy: a screening test for later life cardiovascular disease. Womens Health Issues.

[19] Bellamy L, Casas J-P, Hingorani AD, Williams DJ (2007). Pre-eclampsia and risk of cardiovascular disease and cancer in later life: systematic review and meta-analysisee comment. BMJ.

[20] Melchiorre K, Thilaganathan B, Giorgione V, Ridder A, Memmo A, Khalil A (2020). Hypertensive disorders of pregnancy and future cardiovascular health. Frontiers in Cardiovascular Medicine.

[21] Catov JM, Newman AB, Roberts JM (2007). Preterm delivery and later maternal cardiovascular disease risk. Epidemiology.

[22] Ray JG, Vermeulen MJ, Schull MJ, Redelmeier DA (2005). Cardiovascular health after maternal placental syndromes (CHAMPS): population-based retrospective cohort study. Lancet.

[23] Ness RB, Harris T, Cobb J (1993). Number of pregnancies and the subsequent risk of cardiovascular disease. N Engl J Med.

[24] Parikh NI, Cnattingius S, Dickman PW, Mittleman MA, Ludvigsson JF, Ingelsson E (2010). Parity and risk of later-life maternal cardiovascular disease. Am Heart J.

[25] Clapp JF, Capeless E (1997). Cardiovascular function before, during, and after the first and subsequent pregnancies. Am J Cardiol.

[26] Benschop L, Schalekamp-Timmermans S, Broere-Brown ZA (2019). Placental growth factor as an indicator of maternal cardiovascular risk after pregnancy. Circulation.

[27] Irgens HU, Reisaeter L, Irgens LM, Lie RT (2001). Long term mortality of mothers and fathers after pre-eclampsia: population based cohort study. Br Med J.

[28] Haug EB, Horn J, Markovitz AR (2018). Life course trajectories of cardiovascular risk factors in women with and without hypertensive disorders in first pregnancy: the HUNT study in Norway. Journal of the American Heart Association.

[29] Kaufman L, Rousseeuw PJ (1990). Finding groups in data: an introduction to cluster analysis.

[30] Roberts JM, Rich-Edwards JW, McElrath TF, Garmire L, Myatt L, Global PC (2021). Subtypes of preeclampsia: recognition and determining clinical usefulness. Hypertension.

[31] McElrath TF, Cantonwine DE, Gray KJ (2020). Late first trimester circulating microparticle proteins predict the risk of preeclampsia < 35 weeks and suggest phenotypic differences among affected cases. Sci Rep.

[32] Ramey SL, Schafer P, DeClerque JL (2015). The Preconception Stress and Resiliency Pathways Model: a multi-level framework on maternal, paternal, and child health disparities derived by community-based participatory research. Matern Child Health J.

[33] LeBrasseur NK (2017). Physical resilience: opportunities and challenges in translation. J Gerontol A Biol Sci Med Sci.

[34] Roberts JM, Escudero C (2012). The placenta in preeclampsia. Pregnancy hypertension.

[35] Catov JM, Bodnar LM, Ness RB, Barron SJ, Roberts JM (2007). Inflammation and dyslipidemia related to risk of spontaneous preterm birth. Am J Epidemiol.

[36] Ray JG (2006). Metabolic syndrome and higher risk of maternal placental syndromes and cardiovascular disease. Drug Dev Res.

[37] Roberts JM, Bell MJ (2013). If we know so much about preeclampsia, why haven’t we cured the disease?. J Reprod Immunol.

[38] Redman CWG, Staff AC, Roberts JM (2020). Syncytiotrophoblast stress in preeclampsia: the convergence point for multiple pathways. Am J Obstet Gynecol.

[39] Ness RB, Roberts JM (1996). Heterogeneous causes constituting the single syndrome of preeclampsia: a hypothesis and its implications [Review]. Am J Obstet Gynecol.

[40] Chen Y, Zhang Z, Wu C (2020). AI-PLAX: AI-based placental assessment and examination using photos. Comput Med Imaging Graph.

[41] Bibault J-E, Giraud P, Burgun A (2016). Big Data and machine learning in radiation oncology: state of the art and future prospects. Cancer Lett.

[42] Madabhushi A, Lee G (2016). Image analysis and machine learning in digital pathology: challenges and opportunities. Med Image Anal.

[43] Yala A, Barzilay R, Salama L (2017). Using machine learning to parse breast pathology reports. Breast Cancer Res Treat.

[44] Coudray N, Ocampo PS, Sakellaropoulos T (2018). Classification and mutation prediction from non-small cell lung cancer histopathology images using deep learning. Nat Med.

[45] Chen P, Pan C (2018). Diabetes classification model based on boosting algorithms. BMC Bioinformatics.

[46] Spänig S, Mohsen S, Hattab G, Hauschild A-C, Heider D (2021). A large-scale comparative study on peptide encodings for biomedical classification. NAR genomics and bioinformatics.

[47] Libbrecht MW, Noble WS (2015). Machine learning applications in genetics and genomics. Nat Rev Genet.

[48] Heider D, Dybowski JN, Wilms C, Hoffmann D. A simple structure-based model for the prediction of HIV-1 co-receptor tropism. BioData Mining*.* 2014;7, 1–11, 64. 10.1186/1756-0381-7-14 PMC4124776 25120583

[49] Lengauer T, Sing T (2006). Bioinformatics-assisted anti-HIV therapy. Nat Rev Microb.

[50] Breiman L (2001). Random Forests. Mach Learn.

[51] Neumann U, Genze N, Heider D (2017). EFS: an ensemble feature selection tool implemented as R-package and web-application. BioData Mining.

[52] Schwarz J, Heider D (2019). GUESS: projecting machine learning scores to well-calibrated probability estimates for clinical decision-making. Bioinformatics (Oxford, England).

[53] Beinecke J, Heider D (2021). Gaussian noise up-sampling is better suited than SMOTE and ADASYN for clinical decision making. BioData Mining.

[54] Gandouz M, Holzmann H, Heider D (2021). Machine learning with asymmetric abstention for biomedical decision-making. BMC Med Inform Decis Mak.

[55] Bodnar LM, Cartus AR, Kirkpatrick SI (2020). Machine learning as a strategy to account for dietary synergy: an illustration based on dietary intake and adverse pregnancy outcomes. Am J Clin Nutr.

[56] Jhee JH, Lee S, Park Y (2019). Prediction model development of late-onset preeclampsia using machine learning-based methods. PLoS One.

[57] Escobar GJ, Soltesz L, Schuler A, Niki H, Malenica I, Lee C (2021). Prediction of obstetrical and fetal complications using automated electronic health record data. Am J Obstet Gynecol..

[58] Sufriyana H, Wu YW, Su EC (2020). Artificial intelligence-assisted prediction of preeclampsia: development and external validation of a nationwide health insurance dataset of the BPJS Kesehatan in Indonesia. EBioMedicine.

[59] Roberts JM, Mascalzoni D, Ness RB, Poston L, Global PC (2016). Collaboration to understand complex diseases: preeclampsia and adverse pregnancy outcomes. Hypertension.

[60] Myatt L, Redman CW, Staff AC (2014). Strategy for standardization of preeclampsia research study design. Hypertension.

[61] Duffy JMN, Cairns AE, Magee LA (2020). Standardising definitions for the pre-eclampsia core outcome set: a consensus development study. Pregnancy hypertension.

[62] Duffy J, Cairns AE, Richards-Doran D (2020). A core outcome set for pre-eclampsia research: an international consensus development study. BJOG: An International Journal of Obstetrics & Gynaecology.

[63] Myers JE, Myatt L, Roberts JM, Redman C (2019). Pregnancy C Global COLLECT, a collaborative database for pregnancy and placental research studies worldwide. BJOG An International Journal of Obstetrics & Gynaecology..

[64] Ness R. Innovation Generation. How to produce creative and useful scientific ideas. *.* New York, NY: Oxford University Press; 2012.

[65] Stekhoven DJ, Bühlmann P (2012). MissForest–non-parametric missing value imputation for mixed-type data. Bioinformatics (Oxford, England).

