# Supplementary material for: Vision for Improving Pregnancy Health: Innovation and the Future of Pregnancy Research
Source: Reprod Sci. 2022 May 9;29(10):2908–20. doi: 10.1007/s43032-022-00951-w (PMC9537127; doi:10.1007/s43032-022-00951-w)
Supplement: Supplementary file 1 — Supplementary file1 (DOCX 16 KB) [file 43032_2022_951_MOESM1_ESM.docx]

**Vision for improving pregnancy health:** **Innovation and the Future of Pregnancy Research**

James M. Roberts MD.

Dominik Heider PhD.

Lina Bergman MD, PhD

Kent L. Thornburg, MS, PhD.

**Corresponding Author**

James M. Roberts MD. Magee-Womens Research Institute, Department of Obstetrics Gynecology and Reproductive Sciences, Epidemiology and Clinical and Translational Research University of Pittsburgh, Pittsburgh Pa. USA.

jroberts@mwri.magee.edu

**Supplemental Material**

In machine learning applications, the data is typically provided as tables (e.g., clinical or demographical data), images (e.g., MRT, CT, etc.), or as sequences (e.g., omics data or text). There are many studies describing the preprocessing of image or sequence data, which is beyond the scope of this article. Thus, we focus on clinical data, typically represented as a table with n rows (i.e., patients) and p columns (i.e., the clinical parameters, such as blood parameters, BMI, age, sex, etc.). After setting up the data in tabular format, one needs to clean the data, i.e., one must make sure that there are no missing values, only unique samples, no outliers, etc. Finally, each row in the table represents one patient and each column represents  a feature, for instance, a specific blood parameter of a given patient in the dataset. The input vector is then propagated through the network, i.e., through the hidden layers, to the final output layer, where the outputs are generated. Typically, there is just a single value between 0 and 1, with 1 representing a positive sample, e.g., in diagnostics a cancer patient, while 0 represents a healthy control. The propagation through the network is basically based on matrix multiplications, where each output of a neuron is multiplied with a certain weight, wij, and then used as an input for the subsequent neurons. This procedure describes the usage of a trained neural network, i.e., when it is used for classification of new, unseen samples. However, prior to usage of a network, a network needs to be trained with training samples. These training samples are used to adjust the weights, wij ,by a procedure called backpropagation, which tries to minimize the error at each neuron.

While it is essential that the training data is complete and preprocessed, completeness is not always guaranteed in clinical settings, e.g., some parameters have not been measured for a given patient or are simply unknown. For missing data, different strategies can be applied. Patients with many missing data could be removed from the training data. While this strategy is easy and straightforward, it reduces the available data for training. This is particularly problematic as clinical datasets are often limited in size and thus, every sample is important. Another strategy is to remove a certain parameter if too many samples lack this parameter. Again, this strategy is easy, however, might affect subsequent model building, in particular if the parameter has a direct or indirect association with the disease. An example here is age, which is often associated with different diseases or clinical outcome. A third strategy is imputation, which tries to predict the missing values. These imputation techniques can be simple, thus as using the expectation value, e.g., the mean, or rather complex, e.g., by using another machine learning model to predict the missing values from the other available parameters. A good example of such a model is MissForest,[65] (which uses a random forest to predict missing values). It should be mentioned, however, that data imputation techniques should be applied with care and only on the training data, and must not been used on the validation data, i.e., on the data that is used to evaluate the performance of a trained model on unseen data.
